# Making sense of “superbugs” on YouTube: A storytelling approach

**DOI:** 10.1177/0963662521989251

**Published:** 2021-02-11

**Authors:** Monika Djerf-Pierre, Mia Lindgren

**Affiliations:** University of Gothenburg, Sweden; Swinburne University of Technology, Australia

**Keywords:** antibiotic resistance, genre analysis, health communication, journalism, science communication, storytelling, superbugs, YouTube

## Abstract

Antimicrobial resistance is one of the greatest challenges facing the world. With the rapid growth of social media, YouTube has become an influential social media platform providing publics with expert health knowledge. This article explores how antimicrobial resistance is communicated on YouTube. Drawing on qualitative media analyses of the most viewed YouTube videos 2016–2020, we identify seven different genres and two main storytelling approaches, personalized and fictionalized storytelling, used to make sense of antimicrobial resistance and its complexities. The study contributes new knowledge about YouTube as a platform for health communication and the types of videos about antimicrobial resistance that gets most traffic. This is useful, not the least for public health experts working to improve communication strategies that target hard-to-reach media publics.

## 1. Introduction

The threat of so-called superbugs is a major challenge facing humanity in the twenty-first century (World Health Organization (WHO), 2015). Systematic misuse and overuse of antibiotics as medicine and in food production have created antimicrobial resistance (AMR), making antibiotics no longer universally effective cures for diseases. A key objective in WHO’s Global Action Plan (2015) is to increase awareness, knowledge, and engagement from broad sections of society, including the general public, to improve understanding of AMR.

Media plays a central role in health communication by circulating information from public health experts (Walsh-Childers, 2016) and social media increasingly provides platforms for participatory culture with content production by the public (Davis et al., 2018). YouTube is an influential social media platform, with a transnational reach of two billion monthly users in all regions of the world. In 2020, YouTube was available in 100 countries, and in 80 language versions.^1^ This study centers on the English language version of YouTube with content searched from Australia. Based on qualitative media analysis of the 41 most viewed YouTube videos on “antimicrobial resistance,” “antibiotic resistance,” and “superbugs” in 2016, 2018, and 2020, we explore how storytelling is used to communicate AMR in the variety of genres that exist on YouTube.

The aim of the study is to further our understanding of how AMR is communicated and made sense of on YouTube. This informs efforts to increase public awareness and understanding of this complex issue in at least two ways. First, YouTube constitutes a significant part of the information environment in contemporary mediatized societies, in particular for the younger generations who do not regularly read, watch, or listen to traditional news media. From a health communication perspective, it is important to know what kind of AMR information circulates in social media environments. Second, by looking deeper into the storytelling approaches employed in the most viewed videos on YouTube, we may identify the type of stories and ways of communicating AMR that actually succeeds in engaging audiences. It both contributes new knowledge about YouTube as a platform for specific health communication and the types of videos about AMR that get most traffic. This is useful, not the least for public health experts struggling to devise communication strategies that target hard-to-reach media publics.

### Health communication on YouTube

YouTube is a platform, an infrastructure which provides opportunities for a range of producers to publish their material. It is often conceived of as a place for non-professional producers (amateurs) to create and upload their content, but studies show that content originating from corporate users, such as big media companies in film, music or television, or web-TV companies, dominates on YouTube (Burgess and Green, 2009, 2018). Welbourne and Grant’s (2015) study of the characteristics of the most popular videos about science on YouTube argues that professionally generated content is still superior in number; however, user-generated content is significantly more popular in terms of views.

YouTube’s extensive reach particularly among young audiences has prompted public health experts and educators to use YouTube to reach new audiences. National health agencies, international organizations such as the WHO, and nongovernmental organizations (NGOs) upload videos on YouTube in the hope of educating, engaging, and promoting action among various publics. Patients and individual members of the public also turn to YouTube to communicate, discuss, and create online communities around health issues.

These features have inspired scholars to examine YouTube videos on a wide range of topics. Previous health research on YouTube mainly comprises large-scale quantitative content analyses of health-related videos. Scholarly attention has been on evaluating YouTube as a source of health information, focusing on factual accuracy and quality, with recent studies echoing health professionals’ and policy-makers’ fears of social media spreading misinformation to the public (Bora et al., 2018; Briones et al., 2012; Drozd et al., 2018; Leong et al., 2018, 2019; Pandey et al., 2010; Sahin et al., 2019). In a review of the literature, Madathil et al. (2015) concluded that although YouTube holds a vast amount of data pertaining to health care, some of this information is misleading or incorrect. Despite the growing interest in YouTube, there is limited scholarship examining YouTube videos about AMR. Basch et al’s (2018) quantitative content analysis of videos about *Clostridium difficile* focuses on information quality. Djerf-Pierre et al.’s (2019) study of forms of audience engagement to YouTube videos about AMR identified seven main forms of high-level engagement, including expressions of emotions, blame, and calls for action. This study shows that journalism plays an important role on YouTube by generating audience discussions about social and political accountability. Qualitative research on YouTube is less prevalent and mostly focuses on YouTube as a platform for digital storytelling. Lambert (2018) describes digital storytelling as short autobiographical videos created by amateurs rather than professionals. Studies show how patients use YouTube videos to tell their own stories and lived experiences of illness, treatments, medical care, and recovery (cf. Chou et al., 2011).

This study thus fills a gap in research on health-related issues on YouTube, by identifying and categorizing the variety of genres (and producers) that engage audiences with stories on AMR on the YouTube platform. By mapping what content is featured, and the ways the most popular AMR stories are told, the study will be of interest to health practitioners and communicators, and scholars from different fields who are interested in science and health communication. Our primary interest is not to examine whether scientific knowledge about AMR is accurately reproduced, or to scrutinize the factual correctness of the content, which is what most health-related studies on YouTube do. We are also not focusing solely on the autobiographical content or uploads by ordinary/amateur users on YouTube. Instead, we take a storytelling approach, drawing from narrative media studies, to examine how AMR—colloquially referred to as “superbugs”—is made sense of in the multitude of genres that circulate on YouTube.

### Making sense of AMR through storytelling

The main rationale for analyzing the YouTube videos through a storytelling framework is the fundamental importance of storytelling for creating and circulating social and cultural meanings in society (Bruner, 1991; Lambert, 2018; Squire et al., 2014). Humans use stories to communicate and make sense of the world around them, but stories also tell us something about the social conditions and relations of the society that produces them. The AMR issue rests heavily on science and expert medical knowledge, and we are specifically interested in how this complex issue is made sense of in and through the stories presented in videos on YouTube (Davis et al., 2018; Djerf-Pierre et al., 2019; cf. Moscovici, 2000).

Although all videos on YouTube are unique, there are obvious recurring features; patterns in the way stories are told that unites different producers and productions on YouTube. From a media analysis perspective, storytelling and genre are closely interlinked; storytelling in the media always relates to and draws from media genres, and “stories and their worlds are crucially shaped by the affordances and limitations of the media in which they are realized.” (Ryan and Thon, 2014: 2). Genres are thus pivotal to all media storytelling, be it on film, video, radio, television, or online.

#### Identifying genres on YouTube

Genres can be understood as families of “texts” at the most basic level; groups of media artifacts that share specific conventional features. These conventions are constituted through “narrative structure or plot structure, rhetoric and discursive positions, as well as more immediate stylistic features like layout, design and size. Some features are genre-specific, whereas others appear in a wide range of genres” (Lüders et al., 2010: 953). The narrative construction of communication and techniques used to produce stories are thus key features of genres.

Genres evolve over time as conventions become established and recognized by both audiences and producers. How a story (in this case in a YouTube video) is presented to the audience will thus influence how it is interpreted and understood. A video presented as “news” or “current affairs” creates other expectations for the viewer than, for example, an “advertisement.” All genre categorization must therefore consider the “industrial origin” of the video (Burgess and Green, 2009: 91), the producers, and their goals and purposes. The *communicative purpose of the video*, as revealed by how the video is presented to the viewer on YouTube, is therefore an essential part of a genre analysis.

A key feature of YouTube, however, is its multitude and variability of content, and as Burgess and Green (2018) state, it has become an even more unstable object of study over time. In many ways, YouTube has disrupted media production conventions (Uricchio, 2009: 35), signifying “the very epitome of digital culture” (Snickars and Vonderau, 2009: 11). In a 5-year study of YouTube videos, Kavoori (2015) found there were no easy ways to categorize YouTube content or define its core genres. However, the study concluded that “youth” and “popular culture” were common elements in the most popular videos. YouTube is characterized both by hybridization and intermediality. Hybridization entails the mixing of genres and media forms that evolve when older and newer “media logics” interact (Chadwick, 2017: xi). Intermediality entails that “texts of a given medium send tendrils toward other media” (Ryan and Thon, 2014: 10). This could include a media product that imitates conventions from other media or texts, or that it explicitly or implicitly references other media objects.

YouTube is thus not “a medium” or even “a social medium.” It is a content platform featuring both native content and videos republished from other publications, such as public and commercial broadcasters and production companies. With porous boundaries, it is challenging to neatly sort videos into defined and established genres. Rather, the hybridized contents display frequent overlap between genre conventions and the experimentation with forms and formats resulting in cross-genre use of storytelling devices.

To do a genre analysis of YouTube is therefore inherently tricky. Our pragmatic approach to categorizing genres thus begins with how the video is presented on the website, its industrial origin, and the producer’s stated communicative purpose. We then trace the genre conventions the various videos “draw from,” referencing intermediality (Ryan and Thon, 2014), rather than simply pigeonholing all videos into conventional genre containers.

#### Identifying storytelling elements in and between genres

Above all, a storytelling perspective directs attention to the narrative aspects of the video genres. Narrative storytelling is, for instance, commonly used in television news as packaged stories following a chronological order can be seen to create interest, suspense, and engagement for the audience (Ekström, 2000). The concept of narrative is, however, used by many disciplines, and there is no definition universally agreed on by researchers (Hinyard and Kreuter, 2007; Ryan and Thon, 2014; Squire et al., 2014). Although some YouTube videos present a cohesive and coherent narrative with an identifiable plotline with beginning, middle, and end, with actions, purposeful characters/actors, conflicts, and resolutions (Hinyard and Kreuter, 2007), other videos are but assemblages, a bricolage, of narrative elements (Manovich, 2008).

An obvious starting point for an analysis of the narrative aspects of YouTube storytelling is to detect if there is a discernible plot and identifiable characters or actors in the YouTube videos. The visual aspects are also crucial for constructing meaning in visual media, and the second analytic lens puts focus on the imagery used and how the visual devises are employed. The script and the use of language, including key metaphors (Charteris-Black, 2004; Höijer, 2011; Maasen and Weingart, 2000), constitute a third analytic lens, exploring how AMR is talked about in the videos. Through these three analytical lenses, we will look for common traits used across many stories and genres.

Drawing from this framework, our study asks,

RQ1: What genres are used to communicate AMR on YouTube?
RQ2: What storytelling elements are used to communicate AMR in the different genres?

## 2. Method and data

YouTube materials on AMR are extensive and diverse and therefore present considerable challenges for sampling and analysis. We adopted a purposive selection criterion adapted from qualitative research that comprised (1) selecting the most frequently viewed videos, (2) including those on the topic of AMR, (3) including a large sample of videos without creating an unworkable data set unsuitable for an in-depth qualitative media analysis.

The searches were conducted from Australia using the English-Australian version of YouTube. From the outset, two researchers conducted searches to compare the hits received. Another parallel search with the same English search terms was also conducted from a Scandinavian country and it yielded similar website hits. The (largely unknown) algorithms that YouTube employ to determine the search results for individual users will impact the search results even when using the same search terms. To partially alleviate this problem, we sorted the searches to receive the “most viewed” videos rather than the most “relevant” (which is the default and also more susceptible to algorithm bias). The data collection took place across three time periods: between 20 March and 6 April 2016, 20 February and 12 March 2018, and 28 January and 30 January 2020. During each period, we conducted searches for videos about AMR on YouTube, using three different search terms (see Appendix for a list of all videos in the study; the YouTube search function only displayed total hits in 2016 and 2018 and not in 2020):

1. antibiotic resistance—36,000 hits 2016; 129,000 hits 2018;
2. superbug*—34,000 hits 2016; 63,200 hits 2018;
3. antimicrobial resistance—8000 hits 2016; 28,500 hits 2018.

Each search was sorted according to the number of views recorded on YouTube. The first 30 items from the three searches each year, with individual YouTube pages and supplementary material, were saved. This method provided an overview of the most frequently viewed material on YouTube about AMR. Then the YouTube videos for the 10 most viewed items from each search were selected for further analysis. Several of the videos about AMR turned up in two or more of the searches and are thus discounted as duplicates (Table 1).

**Table 1.** Genre categorization of the most viewed videos about antimicrobial resistance 2016–2020 (number of unique videos).

Genres	Total
Popular science	13
*Science feature (animated, cartoon film)—sub-genre*	*5*
*Science show—sub-genre*	*5*
*TED-Talk—sub-genre*	*1*
Journalism	9
*Documentary/current affairs—sub-genre*	*7*
*Online news—sub-genre*	*2*
“YouTuber”	7
Curriculum resources	4
Medical entertainment	3
Public health campaign	3
Advertising	2
Total	41

The 30 most viewed videos (in 2016, 2018, and 2020) found using the search strings “antibiotic resistance,” “antimicrobial resistance,” and “superbugs” were categorized. Several videos appeared repeatedly in the searches making the total number of unique videos 41.

We also examined the views over time—from the first search in March 2016 to January 2020—to see whether selected videos from 2016 continued to attract views and to verify their top 10 most viewed ranking. Nine of the 2016 videos were still on the most viewed lists 4 years later. Most had substantially increased number of views (Appendix). All in all, we ended up with a corpus consisting of 41 unique YouTube videos, which represent the most viewed videos on YouTube that address various aspects of AMR in 2016–2020.

We conducted a qualitative content analysis of the 41 videos to identify their narrative construction. First, the researchers watched the videos several times, making detailed descriptions of the audio-visual and textual content, and narrative structures, documenting key features with screenshots. Second, each video was analyzed individually, based on our overarching questions about genres and storytelling elements (see below). Third, we contrasted and compared the videos, to identify common features. To address individual researcher bias, a final analysis was done by the researchers together, where agreements and disagreements over categories and codes were debated and resolved.

We analyzed the videos and systematically recorded the following aspects for each individual video:

1. *Genre, industrial origin, and the communicative purpose of the video.* Does the YouTube description identify a genre, such as video news or advertising? Who are the publishers and producers and what are their communicative goals and purposes? Here we complement the meta-information provided on YouTube with additional desk research of other websites.
2. *Storytelling elements.* How is the story told? We examine (a) the imagery and visual design; (b) script and language; how AMR is talked about in the video, which metaphors are used, and which aspect of the AMR issue is the focus of the story; and (c) the narrative construction, such as plots, chronology, scene setting, dramatic tension, and characters/actors in the story.

Conducting an analysis of the content on YouTube will always be shooting at a moving target. All videos in the study were examined as they appeared on YouTube at the time of study in April 2016, March 2018, and January 2020, respectively. At the time of publication of this article, some videos may have been altered, removed, or rendered inaccessible on YouTube for other reasons.

The study was approved by Monash University Human Research Ethics Committee, project 12816.

## 3. Antimicrobial resistance on YouTube: Analysis

The analysis is sectioned in two parts. The first section presents the identified seven genres, based on the video’s YouTube description and formats (e.g. documentary, advertisement), the producer’s industrial origin, and their communicative purpose such as “educational curriculum resources,” while also taking the content and narrative aspects into account. To make the categorization more nuanced, we operate with distinctions on two levels: genre and sub-genre. The latter share the overall characteristics and communicative purpose of the main genre, but have distinct and identifiable traits that set them apart within the larger group.

In the second section, we explore in greater detail the two main storytelling approaches found across the different genres. Drawing from the analysis of visuals, script/language, and plot/characters in the videos, we identify clusters of elements that are commonly used in a larger group of videos.

### Genres, producers, and communicative purposes

The following section outlines the seven genres identified in the analysis (Table 1):

The first and largest category, *Popular science for public education*, comprises 13 videos, with the sub-genres *Science feature, Science show*, and *TED-Talk*. The producers of the videos often operate with a not-for-profit motive, drawing on different sources of funding, including crowdfunding, patrons, pledges, and partnerships with universities or foundations. Commonly they examine antibiotic resistance by focusing on the biological processes involved. Their shared communicative purpose is to provide public education and entertainment by explaining the science of AMR to a general public in compelling, often fun and accessible ways.

**Figure 1.**
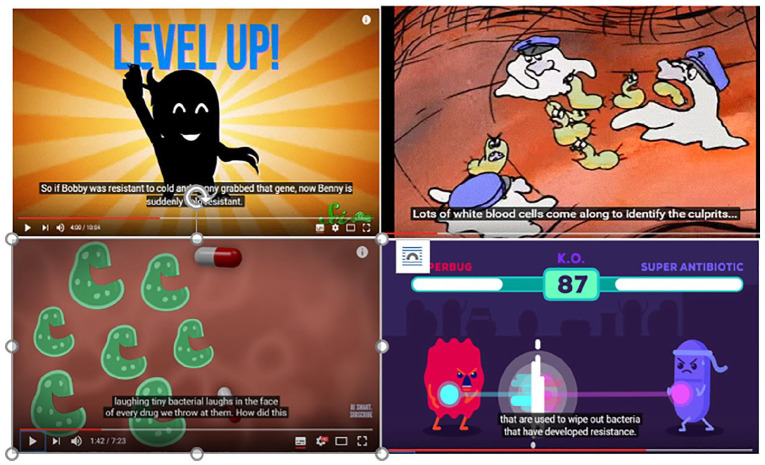
Anthropomorphized bacteria in popular science videos. Screenshots from videos (from top left) 8 (Attack of the superbugs published by SciShow), 33 (Mutations - selection: the bacteria resist by evolutionoflife09), 11 (Rise of the superbugs published by GROSS Science), and 1 (The antibiotic apocalypse explained by Kurzgesagt). See also Appendix for additional information about the videos and the original sources.

The five videos (1, 2, 7, 12, 33) in the sub-genre *Science features* are cartoon-style animated videos with an unseen narrator explaining complex biological processes that result in AMR. Five of the videos can be characterized as *Science shows* (8, 11, 14, 19, 25) presented by young, engaged hosts targeting a young (teen) audience, with videos combining upbeat presentations with cartoon-style animations. Figure 1 show examples of how different cartoons are used in four of the Popular science videos (1, 8, 11, 33).

The one *TED-Talk* (video 28) is a recorded talk to a live audience, where the speaker presents her story about why the world is entering a post-antibiotic era using visual graphics as aid. TED-Talks are produced by TED, an American nonprofit that posts talks online for free distribution on a broad range of subjects under the slogan “ideas worth spreading” (https://www.ted.com/).

The final two videos in the popular science category (3, 30) are two versions of a narrated video recording of an experiment conducted by Harvard University, demonstrating in fast motion how bacteria in a Petri dish develop resistance to extremely high concentrations of antibiotics.

The second largest genre in the sample is *Journalism*, comprising nine videos with the sub-genres *Documentary/current affairs* (4, 9, 13, 21, 24, 34, 41) and *Online news* (38, 40). The documentaries are all long-form journalism with highly developed narratives, produced by professional media production companies (e.g. *Journeyman Pictures, VICE News*) and broadcasters such as the *Australian Broadcasting Corporation* and *Al Jazeera*. YouTube republishes the videos which have all been broadcast elsewhere. All have in common that they examine antibiotic resistance within societal structures, scrutinizing economic and political factors contributing to the problem, such as the Indian pharmaceutical industry or European meat industries, and telling personal stories of people who are victims or otherwise affected by the threat from resistant bacteria.

The two online news native videos are produced for YouTube by for-profit news channels (*WatchMojo.com* and *DNews*) that provide general interest news. They are presented in a mainly factual news reporting style, overlaying stock footage (of doctors, laboratory work, pills and magnified bacteria, close-up images from meat industries and of suffering people in the global south) with a reporter voice-over. The two videos aim to provide the audience with facts and explanations of an issue in a concise and simplified way. The presentation is descriptive with limited scrutiny or analysis, but emphasizing both in script and imagery the urgency and immediate threat of AMR as a global crisis.

The third largest genre with seven videos (11, 18, 20, 32, 35, 37, 39) is produced by *YouTubers.* The videos are made by individuals who host their own YouTube channels, with very different purposes. As a result, the videos are extremely varied in content and form. Commonly, the YouTuber channels do not specifically focus on antibiotic resistance and/or scientific matters; they have a much broader range of content.

Two YouTuber videos consist of edited visuals presented without narration. One is a montage of news reports on tsunamis, earthquakes, pandemics, and superbugs used to signal the imminent apocalypse: introduced with the line “the end of the world, my friends” (10). A second video consists solely of silent footage of Methicillin-resistant *Staphylococcus aureus* (MRSA) skin infections without commentary (32).

Two YouTuber videos have more educational and instructive purposes. Video 20 consists of a voice-over commentary over writing and drawing on a whiteboard, going into scientific details about the differences between gram-positive and gram-negative bacteria. It is similar in style and approach to the videos in the curriculum resources category (below). Another video begins with a YouTuber playing shooter video games, but transfers into an online PowerPoint lecture explaining what antibiotic resistance is and how it can be prevented (37).

The video *Girl Caught New STD “Superbug” and Has Maggots removed from her Vagina?* (35) is the only vlog (or videoblog) among the most viewed AMR videos. Vlogs are videos typically structured around a monologue delivered directly to a webcam. This example is presented by a young woman, whose channel mostly engages with entertainment, music, fashion, and sex/relationships. In this video, she is talking directly to camera, and the viewers, about a video she saw about a woman who had maggots removed from her vagina, something she describes as coming from a new, extremely dangerous STD (“worse than aids, they say”), essentially a “superbug.”

Two of the YouTubers aim more explicitly for social criticism and advocacy. The YouTuber in video 18 usually focuses on videogames, but states that he was compelled to do a video on antibiotic resistance (“So what’s the point of me telling you all this scary shit? Maybe it will help open some of your eyes.”). The video uses stock footage of cartoons and is narrated in an angry and vernacular language blaming “stupid people” and “shitty doctors” and farmers who want “chickens that can bench-press 400 pounds.” Similarly, video 39 is produced by *Vegan Gains*, who uses his site to promote bodybuilding and veganism. In the video, which also displays traits of the vlog but is more advanced in terms of editing and narrative execution, he narrates over stock footage (mostly retrieved from news sources) or does a “piece-to-camera” mimicking a news presenter. Still, the style is argumentative, opinionated, and signals late-night American talk shows with its humorous commentary monologues about the news of the day, often using satire, irony, and sarcasm.

A new genre emerged in the 2018 sample. Three *Public health campaign* videos (15, 17, 26) are published by public organizations and health agencies/groups such as Health Canada and the British government-funded National Health Service (NHS). The storytelling devices are, interestingly enough, the same as in the popular science videos. They use cartoons and animations with humorous scripts, to instruct and motivate viewers to make “healthy decisions”: not to take antibiotics for a flu, wash hands, keep vaccinations up to date, and only take antibiotics as prescribed by a doctor. The short advert from NHS England consists of animated singing and dancing antibiotic pills that exhort the viewer to “always take your doctor’s advice.” Unlike videos in the popular science group, videos in this genre address the individual viewer directly providing explicit instructions and advice.

A further three smaller genres are identified in the analysis. Four videos are *Educational and curriculum resources* (5, 23, 31, 36) aimed at teachers and specific student cohorts, some at advanced level. This genre contains videos made by for-profit companies selling educational resources. Three videos consist of animations that describe specific biological mechanisms and processes, while video 36 is an online lecture drawn on digital whiteboard for high school students. Three videos (6, 29, 22) are characterized as *Medical entertainment* produced for-profit. The videos draw on visceral and spectacular medical content, for example, explicit images of MRSA infections with a high “yuck-factor,” as means to attract audiences. The sensationalist imagery connotes a tabloid style of content, by combining visceral images with a factual, descriptive voice-over narration. The “infection-porn” imagery appeals to audiences who find pleasure in looking at severely infected wounds, pus, and the draining of abscesses. Two videos are classified as *Advertising* (16, 27). They comprise promotional material or brand advertising, produced by companies selling medical equipment and products. Video 16 is a live product demonstration but the IBM advertisement in video 27 is presented as an animated action movie, and tells the story, using monster-puppets, of how their product can successfully fight off AMR.

### Superbug storytelling

Although all videos are unique, with obvious variations in storytelling elements, we aim to identify common storytelling methods and styles. In this section, we look for common traits within and, more importantly, across genres. Two such clusters are found. The first, *fictionalized storytelling*, is dominant in the popular science videos but also in health campaigns and advertising. The second, *personalized storytelling*, is found mainly in journalism but also to some extent in the TED-Talk and in the YouTuber videos.

#### The fictional life of superbugs: Anthropomorphized bacteria and a tale of human tragedy

Although YouTube is said to have significantly changed some existing video production and distribution practices, many videos in our sample draw heavily on narrative forms familiar from television and film but also video games and cartoons. Cartoons and animations are commonly used to explain complex scientific processes and a reoccurring storytelling device is the use of anthromorphized bacteria with human appearances, intentions, and behaviors (Figure 1).

Cartoons and animations are prevalent in all science shows where young and charismatic hosts present programs on camera about superbugs, interspersed with edited segments and humorous stories outlining the history of penicillin or animated antibiotics fighting with superbugs. The TED-Ed video (2) *What causes antibiotic resistance?* uses animated figures to explain how bacteria become resistant, showing an antibiotic pill looking like a cartoon superhero with a cape, fighting and defeating a mean-looking bacterium with threatening sharp teeth. In video 33, animated white blood cells dressed up in police hats are fighting “culprits” looking like small green worms and in video 1 cartoon bacteria and antibiotic pills are engaged in close combat staged as a fighting video game (Figure 1). *The antibiotic apocalypse explained* (1) also uses animated bacteria to explain how resistance is developed. So too, the public health campaign video from NHS (15) employs animated singing antibiotic pills and the video from Health Canada includes cartoon bacteria and anthropomorphized antibiotics (26).

By characterizing bacteria as carrying intent and will (good, evil), and bestowing them human features (eyes, mouths, limbs, and facial expressions displaying emotions or dressing them up in costumes), they are transformed into characters in a plotline. Even without human characters in the story (except as an entity or as a biological vessel affected by bacteria), these videos contain the narrative element of character and agency as bacteria and cells take on human traits and intentions, as in video 11 where cartoon resistant bacteria are shown as laughing when antibiotic pills are thrown at them (Figure 1). They are also involved in sequences of events, including conflicts (fighting with antibiotics) and resolutions (winning or losing). Bacteria as characters are metaphorically described as intelligent and outsmarting humans in the game of evolution: “evolution and antibiotics have teamed up . . .”; “you’ve got to say: well played bacteria” (19); bacteria are “masters of survival” (1).

Video 8, *Attack of the superbugs*, uses anthropomorphization techniques, to help, presumably, young viewers make sense of the issue. Gene transfer is, for instance, described in terms of a popular card game, Pokémon (Figure 1, top left):

They can use something called horizontal gene transfer to swap genetic information like you Swap Pokémon cards.

Conjugation is described as “kind of like sex” and bacteria are personalized by giving them names:

So, let’s say Bobby and Benny E. coli are feeling frisky and Bobby builds a gene passing connection over to Benny. And when they break apart now Benny can do something only Bobby could do before.

IBM’s advertisement *Ninjas vs Superbugs: Adventures in Nanomedicine* (27) also relies on animations and anthropomorphization. It noticeably draws both from monster movies with an animated superbug MRSA balancing on the top of a mountain fighting flying antibiotics, referencing the iconic scene from the movie King Kong on the empire state building, and from fights between supervillains and superheroes in movies and videogames:

MRSA is a superbug super-villain [*animated little red cells, looking terrified, screaming for help*]. It is big and it is bad and we need an entirely new way to beat it. Superbug meet your super-enemy! [*New super swift aircraft-like fighters appear in the form of bur-flowers with ninja masks. The superbug monster gets cut in half and the little red cells cheer*]. (27)

A staple storytelling ingredient in this approach is the prevalent use of war and battle metaphors: antibiotics “kill or neutralize bacteria,” the “deployment of antibiotics” [superhero antibiotic pills flying in battle formation like fighter jets], [antibiotic pill punching bacteria], “booting invaders,” “in the war against superbacteria, de-escalation may sometimes work better than an evolutionary arms race” (all from video 2).

These videos are often loaded with intertextual references anchoring the AMR story in familiar threats and disasters from popular culture, such as games, fantasy, and science-fiction:

What potential disasters keep you up at night? [*host smiles*]. Meteor strikes, super volcanoes, World War Three, World War Z? Those are all pretty scary and we didn’t even mention climate change, but there’s one other immediate, terrifying, scientific problem that rises above the rest. . . . Superbugs. [*lowers his voice, dramatic*]. I’m not talking about giant spiders of Mirkwood or tracker jackers. I’m talking about antibiotic resistant bacteria. Which by the way are *everywhere*. (8)

Videos employing the fictionalized storytelling approach rarely focus on human stories, except for repeated tales of how Alexander Fleming accidentally discovered penicillin. Humans appear as generalized “humans”—addressed as “humanity,” “humans,” “you,” “me,” “us,” and “we.” Humans as a group are metaphorically described as irresponsible, ignorant, and careless: “humans have short memories,” antibiotics are “taken without care,” “humanity is engineering the perfect superbug,” “we are in the process of creating a superbacterium,” “by creating the modern world, we have also built the infrastructure for a dangerous pandemic” (1).

In the fictionalized storytelling approach, the whole of humankind is the main character, with the story plotting humankind’s progress, decline, and (possibly) reform. It is essentially a classic tragedy with apocalyptic references: the “end of the world as we know it” (8). This story of human progress and decline draws on a familiar narrative, a drama ending in the dire and seemingly inevitable post-antibiotic world. This common storyline still often attempts to end on a positive note, positioning science as the “savior” and thus maintaining the myth of the heroic scientist and science as an heroic enterprise (Milne, 1998): “but there is good news . . . scientists are working to stay one step ahead of the bacteria” (2).

#### Personalized storytelling: Lived experiences of superbugs

In the personalized storytelling approach, human experience and struggle constitute the centerpiece with individual persons positioned as central storytelling actors. They convey through lived experiences the impact of superbugs on people, for example, on workers infected in a Danish pig farm (13), sick newborn babies in India (24), or an “ordinary Australian” who nearly died when resistant bacteria spread in his body after a prostate procedure (41). The drama of human suffering and struggle is placed in context of the immediate threat of resistant bacteria, purveyed through the (ever-present) metaphors of doomsday and humans at war with superbugs: “if this goes on, we will see the end of modern medicine” (13); “Pandora’s box may already have been opened” (9); “we have deployed our antibiotic defences far and wide, while the bacteria have kept up their counterattack” (41).

In the TED-Talk (28), US science journalist Maryn McKenna begins her talk with a personal story about her great uncle who died from pre-antibiotic infection when he was 30 years old. The personalized story of the tragic death illustrates both the risks of a world without effective antibiotics and the human impact of AMR, which the audience can identify with:

He was Joe McKenna. He was a young husband and a semi-pro basketball player, and a fireman in New York City. He got hit by a falling device at work and his shoulder got infected . . . he spiked a fever, the fever climbed and climbed. He had what they at the time would call blood-poisoning. The doctors could not do anything . . . He died. (28)

In the video *The rise of India’s superbug* (24), the *Al Jazeera* journalist documents the experiences of two Indian families: one who recently gave birth to a healthy baby girl in a public hospital; the other whose baby died from an infection despite being given multiple antibiotic treatments. As a viewer, we get to know the new family, first in hospital and later at home. The extended focus on one family’s personal story provides an opportunity for connection with humans living with the threat of AMR. It gives a human perspective on a complex and perhaps for many, an abstract health issue. In the *Motherboard’s* video (4) about scientists using phages (virus used as an alternative to antibiotics) to treat drug-resistant bacteria, a young man explains how he has been helped by phage therapy to treat the rare skin disease he suffers from:

My name is Marc Guillonneau and I am 17 years old. My skin peels off every day./. . ./It hurts. Often. /. . ./We tried many different antibiotics. I have lost count of how many I’ve taken. There were so many that I stopped counting. (4)

Personal stories have long been a key feature in journalistic narratives (Wahl-Jorgensen, 2019) as a way to create engagement with audiences who can identify with the plight of others. Humans with their emotions and potential for drama make for compelling storytelling, also providing important opportunities for filming required visuals for TV and video stories. For stories about AMR, this is particularly useful because of visual challenges of stories about bacteria.

In the journalism genre, personalized storytelling features individuals as victims or survivors of resistant bacteria but also the journalists themselves as actors in their own story. In the video *Antibiotic Resistance. Are we creating superbacteria?* (34), the journalist’s daughter, his house, and his interest in gardening feature prominently in the story. In the video, the journalist has his toilet door handle and the surface of his mobile phone tested for bacteria. Some videos with journalists as actors (video 9 about river pollution in India and video 13 investigating overuse of antibiotics in pig farming in Denmark) also draw on storytelling devices that clearly align with established genre conventions of investigative reporting to feature the individual journalist as a “detective” (Campbell, 1987), in their own investigation, searching for clues (facts/evidence) to chase down the culprit (holding those responsible for the problem to account). In the Canadian *CBC* program *Marketplace* (21), the journalist’s search for evidence by lab testing supermarket shrimps provides a narrative spine and a personalized storytelling approach for a story about food security, international food exports, and the use of antibiotics in farming.

## 4. Conclusion

The study identifies seven genres used to communicate AMR on YouTube: Popular Science, Journalism, YouTuber, Curriculum Resources, Medical Entertainment, Public Health Campaigns, and Advertising. The dominance of professionally produced content is obvious. Although the distinction between professional and amateur productions is often blurred, only seven of the videos can be classified as user-generated content, produced by YouTubers with only one vlog video—a format often seen as epitomizing the YouTube culture. Contrary to Welbourne and Grant’s (2015) findings, the professionally produced videos in our sample yielded most views. In the vast majority of the most viewed videos, the communicative purpose is to educate (Popular Science), inform (Journalism), or instruct the public (Public Health Campaigns). In the other samples, the aims and purposes are to sell education (Curriculum Resources), to entertain (Medical Entertainment, some YouTubers), advocacy (some YouTubers), or to sell products (Advertising).

Two main storytelling approaches are used to translate a complex health issue into something that YouTube viewers can relate to and understand. The first, *fictionalized storytelling*, is commonly applied in Popular Science videos but also in Public Health Campaigns and Advertising. It draws on familiar narrative elements from videogames, cartoons, and superhero and monster movies, such as anthropomorphizing bacteria with human appearances, intentions, and behaviors, and staging AMR as a battle between humans/antibiotics and cunning superbugs. The second, *personalized storytelling*, is mainly demonstrated in the journalism genre. Here we find stories with real-life humans as actors sharing their lived experiences, particularly as victims or survivors of resistant bacteria, but also journalists staged as actors in their own story.

These results have several implications for health communication practice as well as research. First, they show that professionally produced videos about AMR on YouTube provide great potential to reach younger publics that are usually hard to engage, at least in the English-speaking parts of the world. Originating from the United States, the platform attracts most users in the United States but also has wide reach in countries such as the United Kingdom, India, Brazil, Thailand, Russia, South Korea, Japan, and Vietnam.^1^ Despite global availability, digital gaps in access and use restricts YouTube’s accessibility in the Global South.

Although we cannot know for certain the actual publics behind the engagement metrics, YouTube’s appeal for the younger generation is well known. Equally certain is, however, that engagement metrics can be manipulated as numerous websites sell fake views, comments, and likes for YouTube channels and videos so some caution against leaning too heavy on metrics is advised (Welbourne and Grant, 2015). YouTube is a platform, a repository, used for many different purposes. We do not know what leads audiences to view a specific video on YouTube in the first place, but it is evident that many organizations use YouTube to host their videos and a significant number of views are generated by funneling viewers to YouTube through links from other sites and platforms (e.g. video 1 in our sample).

The second implication relates to future efforts to produce engaging communications about AMR. The two approaches identified in this study have evidently been successful in engaging audiences, at least with “low-level” engagement such as views (Djerf-Pierre et al., 2019). Both the fictionalized and personalized ways of telling stories about complex AMR issues clearly attract many viewers on YouTube. Both are viable in and of themselves, but a productive way forward could also be cross-fertilization. Public health communication and campaigns can learn much from journalistic storytelling, especially how to engage audiences with personal stories. Journalism, on the other hand, could learn from the popular science videos on how to employ visualizations and humor to offset the scare induced by victims and survivor narratives. In addition, alternative forms of journalism, such as solutions or constructive journalism (see Aitamurto and Varma, 2018 for an overview), could combine journalism’s scrutinizing role with storytelling providing educational material and health instructions to help viewers take actions.

The way storytelling is used to make sense of superbugs on YouTube is furthermore relevant to the efforts to increase public awareness and understanding of this complex issue. The journalism documentaries and current affairs reports engage with critical issues about the societal causes and consequences of AMR and address how resistant bacteria affect people in their everyday lives. On the other hand, the journalistic strategy of highlighting structural issues by in-depth probing of individual cases may result in viewers placing the blame for the AMR problem “somewhere else” (in India, China, or other countries but not “here”). This distancing of viewers risks obscuring the issue as a generalized, global threat that should be a concern for all humans. A closer look at the user comments about this type of videos shows that the personalized storytelling approach indeed has potential for generating both empathy and solidarity, but also risks creating fear and hopelessness or even inciting religious and ethnic resentment, xenophobia, and conspiracy theories (Djerf-Pierre et al., 2019). To be sure, not all engagement in social media is “good” from a civic or democratic point of view.

The fictionalized storytelling about AMR in the Popular Science and Public Health Campaign videos, on the other hand, strives to be socially inclusive by drawing on humankind’s progress and decline narrative, focusing on the relatively uncontroversial biological mechanisms of AMR, thereby avoiding critical perspectives that can offend or alienate. The producers clearly aim for a broad, predominantly young, and reasonably educated audience by employing storytelling techniques familiar to video games and popular movies. This *fictionalization* of the AMR issue—connecting antibiotic resistance to fast-paced action games, superhero movies, and funny cartoons—does, however, have the unintended consequence of diverting the interest from the AMR issue to the fictionalized elements (cartoon figures, popular culture references) in the video per se (Djerf-Pierre et al., 2019). When health communicators turn to YouTube to get a targeted health message across, fictionalized storytelling may stir interest and engagement among younger audiences, but still fail to make reasonable sense of a global crisis.

There are storytelling elements in the videos that transcend even the two main approaches. The omnipresent use of fighting, war, and battle metaphors when telling stories about humans’ relationship with resistant bacteria is something most videos have in common. The prevalent use resonates with much of the dominant rhetoric around AMR using war references such as “the war against superbugs” or the “fight against AMR” (Mendelson et al., 2017: 24). Science and expert medical knowledge about AMR (O’Neill, 2016; WHO, 2015) are also mostly canonized in the videos and the concrete advice provided to address the problem is, when at all present, instructions to maintain hygiene (wash hands) and to “heed your doctor’s advice” when it comes to taking antibiotics. What is largely missing in both approaches to storytelling is human agency (cf. Davis et al., 2018; Marris, 2015). The public is either absent (Popular Science), or posited as ignorant and unwilling to comply (Public Health Campaigns), or portrayed as passive victims/survivors of superbugs (Journalism). AMR communication in all genres should benefit from more deliberative, solutions-based approaches that emphasize human agency and offer concrete solutions to how to relate to antibiotics and resistant bacteria in a practical and sensible way.

## Appendix

**Appendix:** List of the 41 most viewed videos about AMR on YouTube 2016-2020.

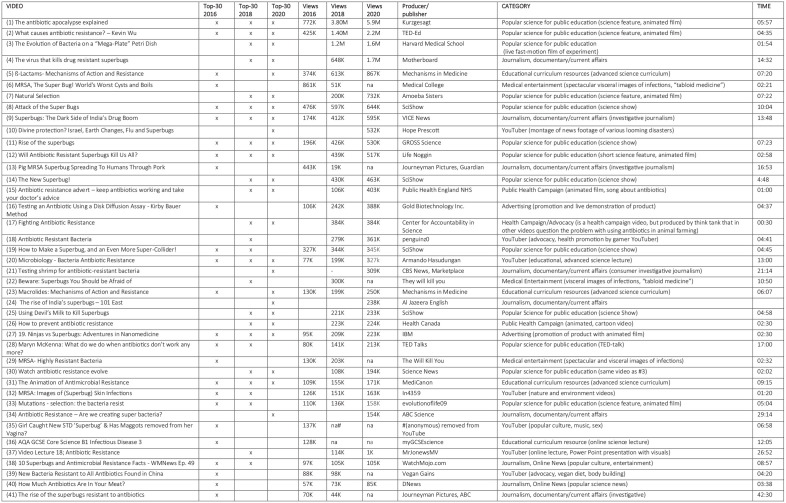

## Notes

1. https://www.youtube.com/about/press/; https://medium.com/@ChannelMeter/youtubes-top-countries-47b0d26dded; https://www.businessofapps.com/data/youtube-statistics/. All accessed 25 August 2020.
